# Identification of Major Rhizobacterial Taxa Affected by a Glyphosate-Tolerant Soybean Line via Shotgun Metagenomic Approach

**DOI:** 10.3390/genes9040214

**Published:** 2018-04-16

**Authors:** Gui-Hua Lu, Xiao-Mei Hua, Li Liang, Zhong-Ling Wen, Mei-Hang Du, Fan-Fan Meng, Yan-Jun Pang, Jin-Liang Qi, Cheng-Yi Tang, Yong-Hua Yang

**Affiliations:** 1 Institute for Plant Molecular Biology, State Key Laboratory of Pharmaceutical Biotechnology, School of Life Sciences, Nanjing University, Nanjing 210023, China; guihua.lu@nju.edu.cn (G.-H.L.); liangliyj@163.com (L.L.); dg1730028@smail.nju.edu.cn (Z.-L.W.); 18851826162@163.com (M.-H.D.); pangyj@nju.edu.cn (Y.-J.P.); 2 Jiangsu Collaborative Innovation Center for Modern Crop Production, Nanjing Agricultural University, Nanjing 210095, China; 3 State Environmental Protection Key Laboratory of Soil Environmental Management and Pollution Control, Nanjing Institute of Environmental Sciences, MEP, Nanjing 210042, China; hxm@nies.org; 4 Jilin Academy of Agricultural Sciences, Changchun 130033, China; mengfanfan0720@163.com

**Keywords:** soil, rhizosphere, glyphosate-tolerant soybean, 16S rRNA gene, plant growth-promoting rhizobacteria, shotgun metagenomic approach

## Abstract

The worldwide commercial cultivation of transgenic crops, including glyphosate-tolerant (GT) soybeans, has increased widely during the past 20 years. However, it is accompanied with a growing concern about potential effects of transgenic crops on the soil microbial communities, especially on rhizosphere bacterial communities. Our previous study found that the GT soybean line NZL06-698 (N698) significantly affected rhizosphere bacteria, including some unidentified taxa, through 16S rRNA gene (16S rDNA) V4 region amplicon deep sequencing via Illumina MiSeq. In this study, we performed 16S rDNA V5–V7 region amplicon deep sequencing via Illumina MiSeq and shotgun metagenomic approaches to identify those major taxa. Results of these processes revealed that the species richness and evenness increased in the rhizosphere bacterial communities of N698, the beta diversity of the rhizosphere bacterial communities of N698 was affected, and that certain dominant bacterial phyla and genera were related to N698 compared with its control cultivar Mengdou12. Consistent with our previous findings, this study showed that N698 affects the rhizosphere bacterial communities. In specific, N698 negatively affects *Rahnella*, *Janthinobacterium*, *Stenotrophomonas*, *Sphingomonas* and *Luteibacter* while positively affecting *Arthrobacter*, *Bradyrhizobium*, *Ramlibacter* and *Nitrospira*.

## 1. Introduction

The worldwide commercial cultivation of genetically modified (GM)/transgenic crops has increased widely during the past 20 years [1]. Cumulatively, more than 1 billion hectares of arable land have been used globally for the commercial cultivation of transgenic soybeans, especially glyphosate-tolerant (GT) soybeans [1,2,3]. This increased commercial cultivation of transgenic crops is accompanied by a growing concern about its potential effects on the soil microbial communities, especially on rhizosphere bacterial communities (reviewed in [4,5,6]) that play important roles in promoting plant health and growth [7,8,9].

Previous studies have shown differential results about the impact of transgenic plants on soil microbial communities including rhizobacterial communities. The release of many transgenic plants including certain transgenic soybean cultivars or lines has no significant effects or exerts only minor and transitory effects on soil microbial communities [10,11,12,13] (also reviewed in [4,5,6,14]). In other cases, the release of some transgenic plants significantly affects the composition or diversity of rhizosphere or root-endophytic microbial communities [15,16,17,18] (also reviewed in [4,5,6,14]).

The soil ecosystem is highly complex, and the proportion of unculturable microbes under standard lab conditions is extremely high [19,20]. Thus, culture-independent methods solely or combined with each other or with culture-dependent methods have been increasingly used to investigate the effect of GM/transgenic plants on soil microbiota [14]. High-throughput sequencing (hereinafter, deep sequencing) of 16S rRNA gene (16S rDNA) amplicons and/or shotgun metagenome library via a next-generation sequencing (NGS) platform, e.g., 454 GS FLX pyrosequencing, is a culture-independent but powerful method that has revolutionarily facilitated soil microbiota research [9,21,22,23,24]. It was first applied to study microbial diversity in the deep sea [25] and also has been applied to detect the possible effects of transgenic plants on soil microbiota [11,26,27,28].

Meanwhile, the Illumina GAIIx [29], Illumina Hiseq, and MiSeq platforms [30,31], which are far more cost-effective than 454 GS FLX pyrosequencing, are powerful NGS technologies that have been successfully applied to study highly complicated microbial communities [32,33,34,35,36]. Subsequently, Illumina MiSeq has also been applied to study the effect of some transgenic plants on microbial communities [18,37,38,39].

Recently, shotgun metagenome sequencing (SMS) via a NGS platform combined with bioinformatics tools has been applied to deeply study the microbial community composition, structure, diversity, and/or function in plant fiber incubated in cow rumen [40], different types of soils [32], activated sludge [41], soil across nitrogen gradients [24], human gut [42], a complex biogas microbial sample from the biogas plants [43], the microbial samples of different hot springs [44,45], taproot sample of a sugar beet [46], permafrost soil [47], and other samples [48,49,50]. To the best of published knowledge at the web of science (http://apps.webofknowledge.com, from all Databases, 118,867,781 data limits until 12 January 2018) through searching with the combined topic words of “transgenic/genetically modified” and “metagenome/metagenomic/metagenomics”, the effects of transgenic plants on soil or rhizosphere microbial communities have been rarely studied via SMS [17,51,52].

Our previous study showed via 16S rDNA V4 region amplicon deep sequencing by Illumina MiSeq that the GT soybean line N06-698 (hereinafter, N698) significantly affects the relative abundances of some rhizosphere bacteria. In specific, systematic contrast analysis revealed that the relative abundance of the special but unidentified operational taxonomic unit (OTU) is remarkably less than 132-fold in the rhizospheric soils of the GT line N698 compared with its control cultivar Mengdou12 (hereinafter, MD12) [18]. In the present study, we performed 16S rDNA V5–V7 region amplicon via Illumina MiSeq and shotgun metagenomic approaches to identify major rhizobacterial taxa affected by the GT soybean line N698.

## 2. Materials and Methods

### 2.1. Plant Materials, Field Design and Sampling

The GT soybean line N698 and its control soybean cultivar MD12 used in this study were described in detail in our previous study [18]. In brief, the GT soybean line N698 was bred by crossing the GT soybean line NZL02-92 containing the *CP4-EPSPS* gene to MD12 and then the GT F1 plants continuously backcrossing to MD12 with two times. Additionally, the female and male parents of NZL02-92 were the conventional soybean cultivar Mengdou13 and the derivative strain of the GT transgenic soybean line AG4501 (See also Patent No. is US5998704-A) that was bred by Asgrow seed Company (Kalamazoo, MI, USA) which was merged to Monsanto company (Creve Coeur, MO, USA), respectively.

Field design and sampling were also described in detail in our previous study [18]. In brief, six sampling points per cultivar were available, and at each sampling point, two soybean plants of N698 or MD12 with adhering surrounding soil were dug out and immediately taken to the laboratory. The soil loosely adhered to soybean plant roots was shaken off and mixed as surrounding soils. Then, the soil tightly adhering to the root surface was brushed off as the rhizospheric soil. The rhizospheric soil collected from soybean plant roots from every two sampling points were mixed together as one biological replicate, and three rhizospheric soil samples were stored in a −80 °C freezer.

### 2.2. DNA Extraction from Rhizospheric Soil Samples

The extraction of metagenomic DNA from rhizospheric soil was described in detail in our previous study [18]. In brief, the metagenomic DNA was extracted from approximately 2 × 0.60 g soil of every biological replicate by using the PowerSoil DNA Isolation Kit (MoBio Laboratories Inc., Carlsbad, CA, USA) in duplicate as recommended by the manufacturer’s instructions but with minor modifications.

### 2.3. Analyses of 16S rDNA via Deep Sequencing Amplicons

#### 2.3.1. PCR Amplification of 16S rDNA (V5–V7) and Illumina MiSeq Sequencing

Our strategy is an improved dual-index sequencing approach with paired-end (PE) 250 nt via Illumina MiSeq [53]. The protocol was described in detail in our previous study [18] but with PE 300 nt and the gene-specific primer for amplifying the V5–V7 region of 16S rDNA, which were 799F (5′-AACMGGATTAGATACCCKG-3′) and 1193R (5′-ACGTCATCCCCACCTTCC-3′). The specific primer pair was selected due to the previous study by Bulgarelli et al. [22] and Schlaeppi et al. [54]. Additionally, the qualified metagenomic DNA of each sample was normalized to 30 ng per PCR reaction within a 50 µL volume. 16S rDNA V5-V7 amplicon deep sequencing clean data of 6 samples have been submitted to the NIH Sequence Read Archive (SRA), and the SRA accession is SRP136046.

#### 2.3.2. Operational Taxonomic Unit Selection and Analysis of Species Composition and Abundance

OTU selection and analysis of species composition and abundance were described in detail in our previous study [18] with minor corrections by using the software UPARSE [55] implemented as the cluster_OTU command in USEARCH (v7.0.1090) [55]. Furthermore, the OTU counts in each sample’s library were normalized using rarefaction corresponding to the sample with the least absolute filtered tags at 97% similarity in the group after species annotation was performed and a phylogenetic tree was constructed.

#### 2.3.3. Alpha and Beta Diversity Analyses

Alpha and beta diversity analyses were also described in detail in our previous study [18]. Briefly, principal coordinate analysis (PCoA) was drawn by software R package v3.1.3 (R Development Core Team) to exhibit the differences between the groups according to the matrices of beta diversity distances calculated by QIIME (v1.8.0) [56].

### 2.4. Shotgun Metagenomic Approaches

#### 2.4.1. Construction of Metagenomic DNA Library

Shotgun metagenomic DNA library was constructed in accordance with the manufacturer’s instructions (Illumina, CA, USA) [57] with some minor modifications. In brief, three metagenomic DNA samples each with 0.4 µg DNA taken from MD12 or N698 rhizospheric DNA sample were pooled as one qualified sample for shotgun metagenome sequencing, named MGMRh or MGNRh. Exactly 1.2 µg qualified DNA of MGMRh or MGNRh sample in 80 µL TE buffer was sheared into small fragments less than 600 bp by nebulization. Afterwards, the 3′ end of the phosphorylated blunt DNA fragments was added with an adenine (A) base, and then ligated with Illumina adapter oligo mix. Furthermore, the adapter-modified DNA fragments were magnified by NEB Phusion high-fidelity PCR master mix (New England Biolabs, MA, USA) with 65 °C melting temperature and 12 cycles. Moreover, adapted DNA fragments of 400–600 bp were purified by QIAquick PCR purification kit (Qiagen, Shanghai, China), and then qualified by Agilent 2100 Bioanalyzer (Agilent Technologies, CA, USA) and quantified by ABI StepOnePlus Real-Time PCR System (Applied Biosystem, CA, USA). The PE shotgun metagenomic libraries were constructed with inserted fragment for the MGMRh and MGNRh samples.

#### 2.4.2. Shotgun Metagenome Sequencing

The qualified metagenomic libraries were deeply sequenced with the PE 125 nt strategy via the Illumina HiSeq2500 NGS platform (Illumina, CA, USA) and HiSeq PE Cluster Kit v4 (Illumina, CA, USA) by BGI Tech Solutions Co., Ltd. (Shenzhen, China). Shotgun metagenome sequencing clean data of MGMRh and MGNRh samples have also been submitted to SRA, and the SRA accession is SRP136046 too.

#### 2.4.3. Quality Control and De Novo Metagenome Assembly

Clean data were obtained after sequencing adapters, and reads with ambiguous N base or average base quality score less than 15 were removed from raw data. De novo metagenome assembly was performed with IDBA-UD (v1.1.1) [58] for each sample, and reads were assembled with a series of different *k*-mer size (25–115 bp) in parallel, and then were mapped back to each of the assembled contigs for validation. The best assembly was selected based on contig N50 and mapping rated, and only those contigs that were longer than 500 bp were kept for further analysis.

Metagenomic analysis was also performed by One Codex data platform (hereinafter, One Codex) [59] with cloud-based *k*-mer method, after the compressed FASTQ data, which was appended with .gz, was uploaded to the One Codex platform [59], considering that One Codex is more accurate than either the MG-RAST or the Kraken tools [60].

#### 2.4.4. Taxonomic Assignment

Based on the known sequence database of bacteria, fungi and archaeobacteria retrieved from the nucleotide database of the National Center of Biotechnology Information (NCBI, GenBank Flat File Release 201.0, until 31 May 2014) including 1,099,685 bacterial sequence entries, clean reads of each sample were aligned by SOAPaligner (v2.21, also named as SOAP2) [61]. Then, mapped clean reads were assigned to the corresponding taxon and summarized. Taxonomic assignment was also performed by One Codex based on the One Codex database (hereinafter, One Codex DB) and the NCBI RefSeq Complete Genomes database (hereinafter, RefSeq DB). One Codex DB contains 37,183 microbial genomes until December 2017, including 30,825 bacterial, 5163 viral, 633 fungal, 504 archaeal, and 57 protozoan genomes, which collect 29,063 more genomes than RefSeq DB (https://app.onecodex.com/references). RefSeq DB has 8120 microbial genomes, including 2948 bacterial, 4726 viral, 264 fungal, and 181 archaeal reference and representative genomes downloaded from the NCBI until December 2017 at the One Codex data platform (https://app.onecodex.com/references).

### 2.5. Statistical Analyses

Wilcoxon rank-sum test was used to examine the significance of alpha diversity indices. Metastats [62], described in detail in our previous studies [18,63], was used to obtain the relative abundance differences of microbial communities between groups (groups = 2, samples per group ≥ 3). The obtained *p*-value was adjusted by Benjamini–Hochberg false discovery rate (FDR) [64] correction (function “*p*.adjust” in the stats package of R (v3.1.3)).

## 3. Results

### 3.1. Composition and Structure of Rhizosphere Bacterial Community Revealed by 16S rDNA V5–V7 Amplicon Deep Sequencing

#### 3.1.1. Overall Analysis of 16S rDNA (V5–V7 Hypervariable Region) Amplicon Sequencing Data-Based Illumina MiSeq

A total of 413,978 qualified pairs of clean reads (300 nt average) were obtained with an average of 68,996 (range: 40,515–106,532) per rhizospheric soil sample of the GT soybean line N698 (NRh) and that of its control cultivar MD12 (MRh). Then, 130,813 filtered tags (417 ± 4 nt) at 97% similarity were obtained with an average of 21,802 (range: 13,416–32,720) per sample. Next, the OTU counts were normalized using rarefaction corresponding to the filtered tags of NRh1 (Table S1). Finally, 5144 OTUs except singletons were identified with an average of 857 OTUs per rhizospheric soil sample (Table S1) and were summarized with taxonomic annotation in Table S2. All OTU sequences were shown in File S1.

#### 3.1.2. Alpha Diversity of Bacterial Communities in Rhizospheric Soil

**Figure 1:**
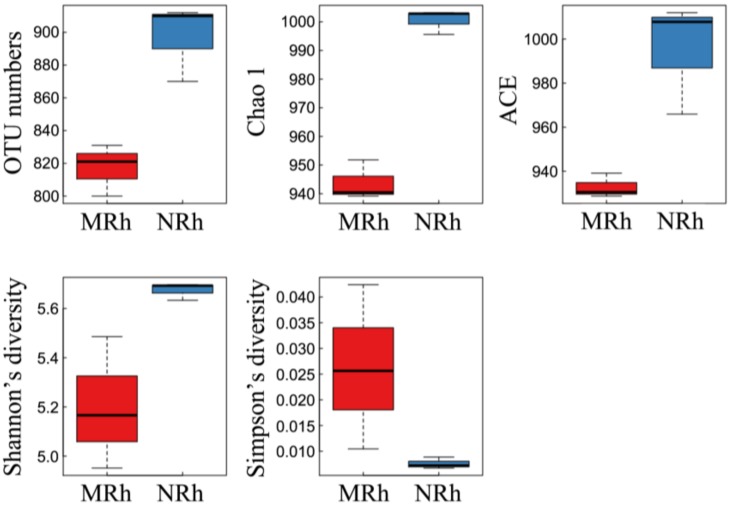
Boxplot analysis of five indices of alpha diversity. Five lines from top to bottom are the maximum value, the third quartile, median, the first quartile, and the minimum value. MRh represents the rhizospheric soil samples of the control soybean cultivar MD12 (n = 3). NRh represents the rhizospheric soil samples of the glyphosate-tolerant (GT) soybean line N698 (n = 3). OTU: operational taxonomical unit; ACE: abundance coverage-based estimator.

The rarefaction curves of the observed OTU number, Chao 1, abundance coverage-based estimator (ACE) and Shannon of rhizospheric soil samples were calculated based on the normalization of OTU counts. All curves nearly reached the saturation plateau (Figure S1), indicating that the sequencing depth included sufficient detectable species in bacterial communities and was sufficient to capture the diversity of bacterial communities in those samples. The mean and standard deviation of five alpha diversity indices of the rhizospheric soil groups were then calculated (Table S3). All p-values of five indices of alpha diversity in Table S3 were higher than 0.05 between the NRh and MRh samples as calculated by Wilcoxon rank-sum test, (Table S3). This result indicates no significant difference in the overall indices of the alpha diversity. However, all five indices of alpha diversity of the rhizospheric soil of N698 were separated from that of MD12 (Figure 1) when boxplot analysis was used to visualize the differences.

#### 3.1.3. Beta Diversity of Bacterial Community in the Rhizosphere Soil

**Figure 2:**
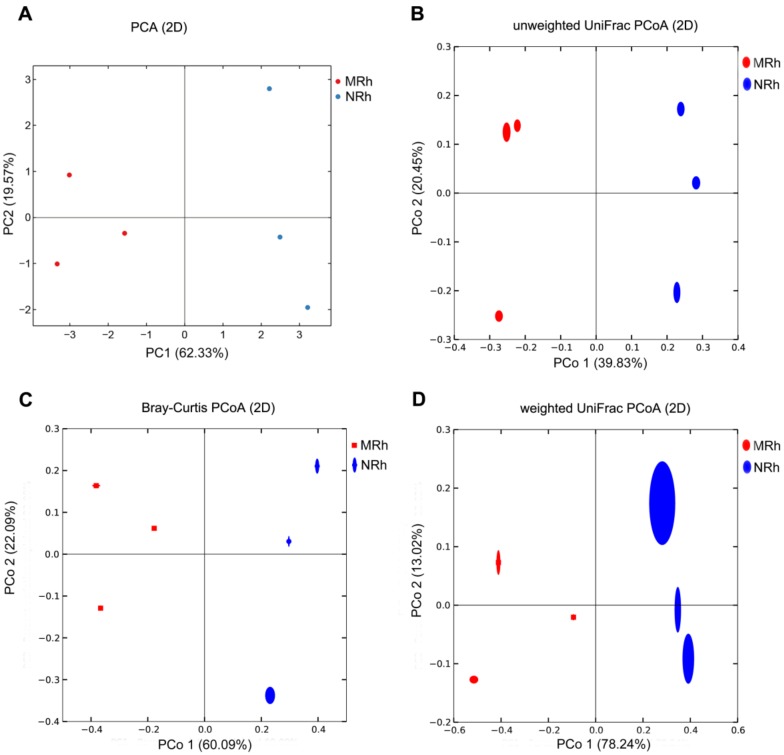
Beta diversity analysis of the rhizosphere bacterial communities between N698 and MD12 (n = 6). Principal component analysis (PCA) based on OTU abundance of bacterial communities (**A**); Numbers in brackets represent contributions of principal components to the total variance; The red and light blue dots represent rhizospheric soil replicates of MD12 (MRh) and those of N698 (NRh), respectively. Principal coordinate analysis (PCoA) based on unweighted UniFrac metrics (**B**), Bray–Curtis metrics (**C**), and weighted UniFrac metrics (**D**); the variance explained by each principal coordinate axis is shown in PCo 1 vs. PCo 2; Red ovals or squares represent MD12, and blue ovals represent N698.

Principal component analysis (PCA) displayed that the bacterial communities in the rhizospheric soil of the transgenic line N698 were distinct from those of MD12 (Figure 2A). Then, phylogenetic beta diversity analysis was performed by PCoA based on the unweighted uniFrac (UUF) and weighted uniFrac (WUF) distance metrics. The bacterial communities in the rhizospheric soil of N698 were distinct from those in the rhizospheric soil of MD12 (Figure 2B,D). Furthermore, taxonomic beta diversity analysis was performed by PCoA based on the Bray–Curtis distance metrics. Here the bacterial communities in the rhizospheric soil of N698 were distinct from those in the rhizospheric soil of MD12 (Figure 2C).

#### 3.1.4. Comparison of Dominant Bacterial Phyla in the Rhizospheric Soil between N698 and MD12

The taxonomic composition and distribution of the rhizospheric soil of N698 and MD12 at the phylum level (Table S4) showed that the most abundant phylum was Proteobacteria, followed by Actinobacteria, Acidobacteria or Gemmatimonadetes, and then by Bacteroidetes or Verrucomicrobia and Firmicutes (Table S4). In addition, the relative abundance of Proteobacteria was significantly (*p* = 0.0163) lower in the rhizospheric soil of N698 than that of MD12 (Table S4).

#### 3.1.5. Comparison of Dominant Bacterial Genera in the Rhizospheric Soil between N698 and MD12

**Figure 3:**
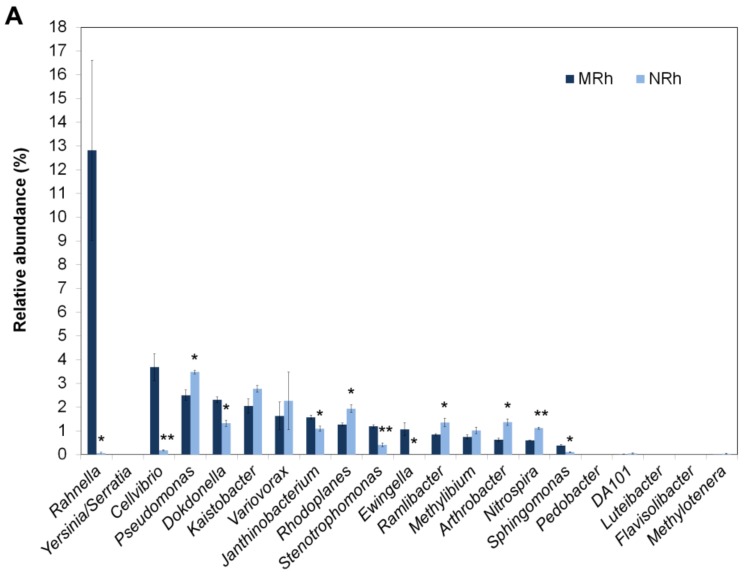

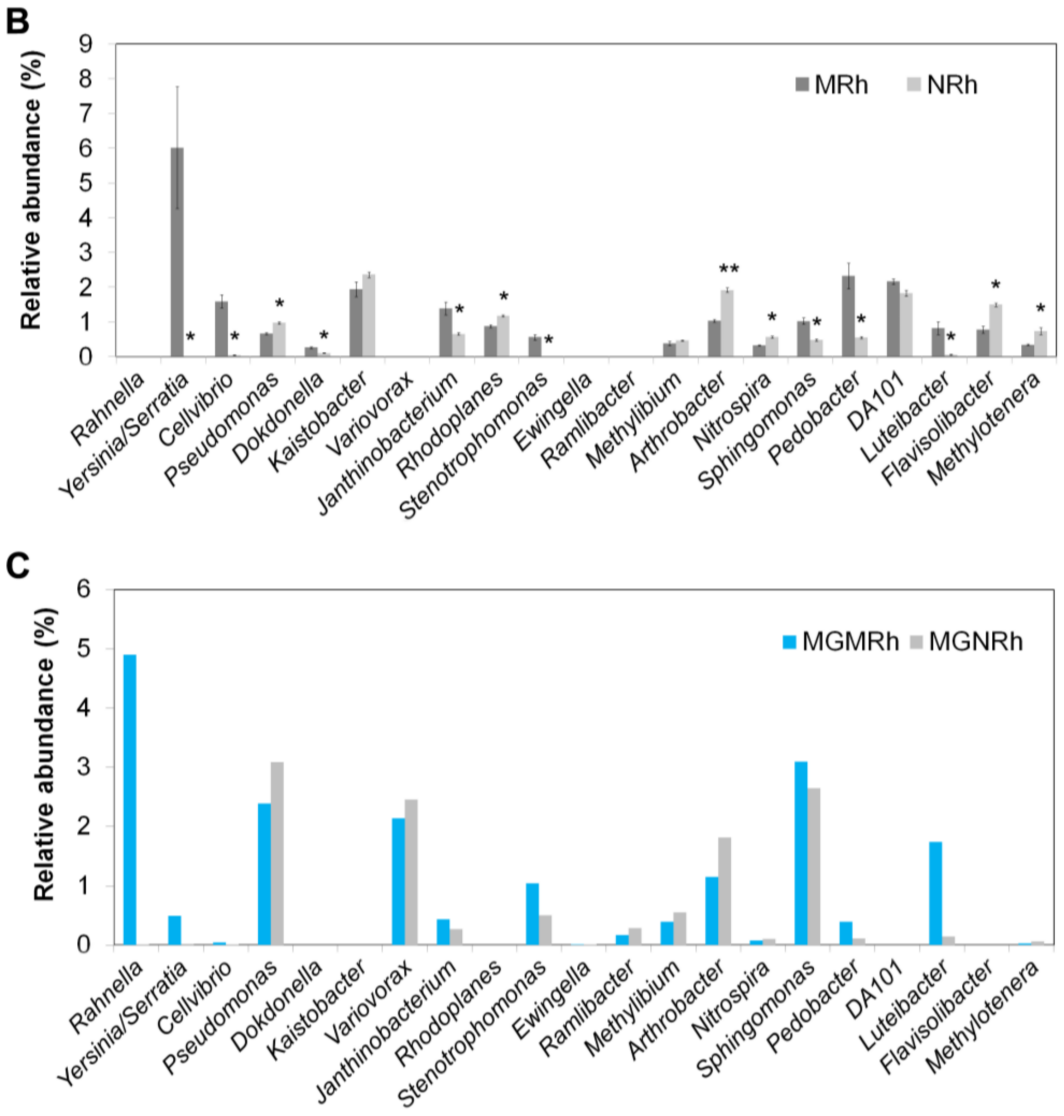
Comparison of the relative abundances of top 10 dominant genera in the rhizospheric soil between N698 and MD12. (**A**) Results revealed by 16S rDNA V5–V7 hypervariable region amplicon deep sequencing. (**B**) Results revealed by 16S rDNA V4 hypervariable region amplicon deep sequencing. (**C**) Results revealed by shotgun metagenomic approaches (analyzed by One Codex). Error bars indicate standard errors; * *p* < 0.05; ** *p* < 0.01. MRh1–3 and NRh1–3 represent three biological rhizospheric soil replicates of MD12 (MRh) and those of N698 (NRh), respectively. MGMRh and MGNRh represent pooled rhizospheric DNA sample of MRh and NRh for shotgun metagenome sequencing, respectively.

A total of 286 genera were detected in the rhizospheric soil of N698 and MD12 (Table S5), and the relative abundances of 36 among 100 characterized genera were significantly (*p* < 0.05) different between the rhizosphere of N698 and MD12 except those unclassified genera (Table S6). Surprisingly, both *Yersinia* and *Serratia* were not found in the normalized OTU table for biom format (Table S2) by 16S rDNA V5–V7 amplicon deep sequencing, although *Yersinia* or *Serratia* was detected by 16S rDNA V4 amplicon deep sequencing. Moreover, we compared the top 30 dominant rhizobacterial genera of N698 and MD12 revealed by 16S rDNA V5–V7 and V4 region amplicon deep sequencing (Table S8) and visualized the top 10 dominant genera in Figure 3A,B. Among those dominant genera, *Rahnella*, *Variovorax*, *Ewingella* and *Ramlibacter* were detected only by 16S rDNA V5–V7 region amplicon deep sequencing. By contrast, *Yersinia*/*Serratia*, *Pedobacter*, *Luteibacter* and *Flavisolibacter* were detected only by 16S rDNA V4 region amplicon deep sequencing (Table S8, Figure 3). We were unable to identify whether the highest abundance OTU is *Rahnella*, *Yersinia* or *Serratia*.

Furthermore, we blasted the 418 bp 16S rDNA V5–V7 region sequence of OTU (File S1) with the highest abundance and found that the OTU should be *Rahnella aquatilis* (Table S7). However, the OTU was 99% similar (416/417 bp) not only to the 16S rDNA of *Rahnella* sp. G7/Y2/Z2-S1, *R*. *aquatilis* strain JS119, but also to the 16S rDNA of *Serratia* sp. THG-CN21 via basic local alignment search tool (BLAST). Furthermore, the OTU was 99% similar (415/417 bp) to the genome fragment of *R*. *aquatilis* CIP 78.65 (ATCC 33071) via BLAST. In our previous study, the 253 bp 16S rDNA V4 region sequence of OTU with the highest abundance in the rhizobacterial community of N698 was 100% similar not only to the 16S rDNA of *Yersinia pestis* CO92, *Yersinia enterocolitica* subsp. *enterocolitica* 8081, and *Serratia proteamaculans* 568, but also to the 16S rDNA of *Rahnella* sp. strain S04, *R*. *aquatilis* strain YHBT21/YHBT11 and to the genome fragment of *Serratia plymuthica* S13 after BLAST [18]. To date, the highest abundance OTU in the present study was still not identified.

Thus, we performed SMS and bioinformatics analysis to deal with this issue.

### 3.2. Composition of Rhizosphere Microbial Communities Revealed by Shotgun Metagenomic Approaches

#### 3.2.1. Statistical Summary of Assembled Shotgun Metagenome Sequencing Data

On average, 58,728,828 clean reads and 7.34 Gbp clean data per sample were generated from SMS (Table S9). The total clean reads of the MGMRh and MGNRh samples were assembled by IDBA-UD (v1.1.1) first, but their mapping rates were only 0.983% and 2.368%, respectively (Table S9). Thus, de novo metagenome assembly of two samples was reperformed with One Codex, which displayed an obvious increase to more than 8.23% of MGMRh or 6.97% of MGNRh in the mapping rates of both samples based on the RefSeq complete genome database (Table S9).

#### 3.2.2. Computation and Comparison of Taxonomic Assignment between MGMRh and MGNRh by SOAPaligner Based on the NCBI Nucleotide Database

Taxonomic assignment was performed via SOAPaligner by aligning clean reads of the MGMRh or MGNRh sample directly to the NCBI nucleotide database, and then mapped clean reads were assigned to the corresponding taxons and summarized (Table S10, sheets 1–6). On one side, the genus with the most mapped read count (316,533 reads) in the MGMRh sample was *Rahnella* (Table 1, sheet 5 of Table S10), and its relative abundance was more than 40% of the total mapped reads and was consistent with the result revealed by 16S rDNA V5–V7 amplicon deep sequencing (Table S6). By contrast, the mapped read count of *Rahnella* in the MGNRh sample was only 385 reads and 0.121% of the total mapped reads (Table 1, sheet 5 of Table S10). This finding is consistent with the result revealed by 16S rDNA V5–V7 amplicon deep sequencing (Table S6). *Serratia* was detected as the top four genus in the MGMRh sample, although it was detected with much less mapped reads in the MGNRh sample. *Yersinia* was also detected but with few mapped reads either in the MGMRh or MGNRh sample (Table 1, sheet 5 of Table S10). Additionally, *Variovorax*, *Ramlibacter*, *Pedobacter*, *Luteibacter* and *Flavisolibacter* were detected by SOAPaligner in either the MGMRh or MGNRh sample (Table 1, sheet 5 of Table S10).

Limited total mapped clean reads were identified via SOAPaligner, i.e., only 790,310 of 59,269,260 clean reads in the MGMGh sample and 318,187 of 58,188,396 clean reads in the MGNRh sample (Table 1, Table S10). Thus, we performed computation and comparison of taxonomic assignment by using another bioinformatics tool, One Codex.

#### 3.2.3. Computation and Comparison of Taxonomic Assignment between MGMRh and MGNRh by One Codex Based on One Codex DB

We uploaded our PE SMS files of the MGMRh and MGNRh samples by using Windows Uploader provided by Minot et al. [59] because we cannot upload our SMS files via One Codex command-line-interface. Basing on One Codex DB, we classified 12.43% of 29,634,630 clean reads in Reads_MGMRh_1.fq.gz (MGMRh_1), 12.29% of 29,634,630 clean reads in Reads_MGMRh_1.fq.gz (MGMRh_2), 10.47% of 29,094,198 clean reads in Reads_MGNRh_1.fq.gz (MGNRh_1), and 10.34% of 29,094,198 clean reads in Reads_MGNRh_1.fq.gz (MGNRh_2) as mixed metagenomic samples at all taxonomic levels by using One Codex (Table S11). On the one hand, the genus with the most mapped read count in the MGMRh sample was *Rahnella*, with a relative abundance of 4.898% of the total mapped reads (Table S12 and Table 1, and Figure 3C). On the other hand, *Rahnella* in the MGNRh sample had only 510 mapped reads, which was only 0.00834% of the total mapped clean reads (Table S12, Table 1). Both results were consistent with those analyzed by SOAPaligner and revealed by 16S rDNA V5–V7 amplicon deep sequencing (Figure 3A,C). *Serratia* was also discovered in the MGMRh sample, but it was detected with much less mapped reads in the MGNRh sample. Meanwhile, *Yersinia* was found with few mapped reads either in the MGMRh or MGNRh sample (Table S12 and Table 1). Furthermore, *Variovorax*, *Ewingella*, *Ramlibacter*, *Pedobacter* and *Luteibacter* were detected by One Codex based on One Codex DB at the genus level in either the MGMRh or MGNRh sample (Table 1 and Table S12).

#### 3.2.4. Computation and Comparison of Taxonomic Assignment between MGMRh and MGNRh by One Codex Based on RefSeq DB

Besides One Codex database, RefSeq DB downloaded from NCBI is available at the One Codex data platform. We further performed taxonomic assignment of MGMRh_1, MGMRh_2, MGNRh_1 and MGNRh_2 files by One Codex based on RefSeq DB. We classified 8.17% of 29,634,630 clean reads in MGMRh_1, 8.06% of 29,634,630 clean reads in MGMRh_2, 6.95% of 29,094,198 clean reads in MGNRh_1, and 6.85% of 29,094,198 clean reads in MGNRh_2 as mixed metagenomic samples at all taxonomic levels by using One Codex (Table S13). The genus with the most mapped read count in the MGMRh sample was *Rahnella*, with a relative abundance of 4.443% of the total mapped reads (Table S14 and Table 1). *Rahnella* in the MGNRh sample had only 364 mapped reads, which was only 0.00897% of the total mapped clean reads. Both results were consistent with the result analyzed by SOAPaligner and by One Codex based on One Codex DB. In addition, *Serratia* was also discovered in the MGMRh sample, but it was detected with much less mapped reads in the MGNRh sample. Meanwhile, *Yersinia* was found with some mapped reads in the MGMRh sample but with much less mapped reads in the MGNRh sample (Table S14 and Table 1). Moreover, *Variovorax*, *Ramlibacter*, *Pedobacter* and *Luteibacter* were detected in either the MGMRh or MGNRh sample, whereas *Ewingella* and *Flavisolibacter* were not detected at the genus level by One Codex based on RefSeq DB (Table 1 and Table S14).

#### 3.2.5. Identification of Rhizobacterial Species of *Rahnella* and *Serratia* by Shotgun Metagenomic Approaches

At the species level, *Rahnella* sp. Y9602 had the most mapped read count in the MGMRh sample, and *R. aquatilis* was one of the top 10 species in the MGMRh sample revealed by SOAPaligner, but *Rahnella* sp. WP5 was undetected. Additionally, *Serratia liquefaciens* was the 3rd top species in the MGMRh sample revealed by SOAPaligner (Table 2, and Table S10 in detail). However, the mapped clean reads of all three species were few in MGNRh sample (Table 2, and Table S10 in detail).

Based on the One Codex DB, *Rahnella* sp. WP5 was identified as the top species with the most mapped read count in the MGMRh sample revealed by One Codex (Table 2, and Table S15 in detail). *R. aquatilis* also belonged to the top 10 species, and *Rahnella* sp. Y9602 was detected with 12,208 mapped clean reads. Meanwhile, *S. liquefaciens* was one of the top 10 species (Table 2, and Table S15 in detail). With regard to the results analyzed by SOAPaligner, the mapped clean reads of all three species were found with few counts in the MGNRh sample (Table 2, and Table S15 in detail).

On the basis of the RefSeq DB, *R. aquatilis* was identified as the top species with the most mapped read count in the MGMRh sample revealed by One Codex (Table 2, and Table S16 in detail). However, not only *Rahnella* sp. WP5 and *Rahnella* sp. Y9602 but also other species of *Rahnella* were undetected. Meanwhile, *S. liquefaciens* was undetected (Table 2, and Table S16 in detail), although some other species of *Serratia* were detected. We suppose that this result might be due to the limited 2948 bacterial genomes in RefSeq DB.

## 4. Discussion

### 4.1. The Impact of the GT Soybean Line N698 on the Rhizobacteria Has Been Confirmed by 16S rDNA V5–V7 Amplicon Deep Sequencing

In the present study, the box plot analysis of all alpha diversity indices (Figure 1) indicated that the species richness and evenness of the bacterial communities were higher in the rhizospheric soil of N698 than that of MD12, but the difference was not statistically significant (Wilcoxon test). Furthermore, PCoA based on WUF, UUF and Bray–Curtis distance metrics indicated that N698 influenced the beta diversity of the rhizospheric soil bacterial community compared with MD12 (Figure 2). Additionally, some bacterial phyla related to N698 are Proteobacteria, Gemmatimonadetes and Planctomycetes (Table S4). Certain bacterial genera related to N698 are *Rahnella*, *Cellvibrio*, *Janthinobacterium*, *Rhodoplanes*, *Stenotrophomonas*, *Arthrobacter*, *Sphingomonas* and *Nitrospira*. (Figure 3A, Table S6). These above results support our previous study on the impact of N698 on the rhizobacteria via 16S rDNA V4 hypervariable region amplicon deep sequencing [18].

### 4.2. Shortcomings and Solutions of 16S rDNA Amplicon Deep Sequencing

16S rDNA amplicon deep sequencing via NGS technologies has vastly facilitated the research of complicated microbial communities. However, it still has major technical shortcomings [65], especially 454 pyrosequencing with low sequencing depth, including biases caused during PCR amplification [66,67,68], chimeric sequences [68], sequencing errors [68,69] and primer mismatches [70].

Recently, amplicon sequencing errors have decreased to 0.02% by using effective sequence analysis pipelines [55,68], and low-error 16S rDNA amplicon deep sequencing approaches have been developed [71]. Moreover, UCHIME has been developed to significantly reduce chimeric DNA sequences (chimeras) in amplicon deep sequencing data [72]. To reduce biases caused by PCR amplification, Schmidt et al. recommended pooling multiple PCR repeats amplified from the same biological replicate DNA [33]. Furthermore, 16S rDNA fragments generated from Illumina-based SMS is a powerful alternative to 16S rDNA amplicons [70]. To evaluate the bias of Illumina-based sequencing of bacterial 16S rDNA amplicon, Kennedy et al. performed PE Illumina sequencing with sufficient depth and found that template concentration exerts a more significant effect on sample profile variability than sample preparation and pooling of multiple PCR amplicons [73].

In addition, the detection limits of 16S rDNA amplicon deep sequencing are low reproducibility and quantitation, especially for beta diversity [74,75]. Therefore, increasing sampling efforts, including sequencing efforts, and the number of sample replicate are the most effective ways to improve technical reproducibility and quantitation [65,75]. Thus, in the present study, an average count per sample with more than 68,996 paired clean reads was near the sequencing number for the desired 90% OTU overlap.

Some inconsistent results of relative abundances at different classifications between 16S rDNA V4 amplicons in our previous study [18] and V5–V7 amplicon deep sequencing in the present study may be attributed not only to the lack of pooling multiple PCR repeats but also to the different hypervariable regions of 16S rDNA. Furthermore, due to short length of 16S rDNA amplicon, it is very difficult to distinguish OTU at the genus level, especially at the species level. Deep sequencing full-length 16S rDNA by third-generation sequencing technologies, PacBio single-molecule real-time (SMRT) sequencing system, is a much effective alternative to overcome 16S rDNA amplicon PCR biases and primer mismatches [76].

### 4.3. Shotgun Metagenome Sequencing via NGS Technology and Bioinformatics Tool

To identify the richest OTU in the present study, we performed SMS via Illumina Hiseq2500 combined bioinformatics tools instead of deep sequencing full-length 16S rDNA because SMS is not affected by PCR biases or chimeras. In addition, SMS is a powerful method not only for analyzing the entire phylogenetic, taxonomic, genetic and functional diversity of microbial communities but also for discovering new genes, regulators and pathways [65].

However, the large datasets generated by NGS platforms, e.g., 454 GS FLX, Illumina HiSeq/MiSeq, and Ion Torrent PGM, require specialized computing hardware and software and need massive computational resources but still produce relatively short contigs in many studies (reviewed in [65]) and the present study by IDBA-UD [58]. In addition to IDBA-UD, numerous bioinformatics approaches, e.g., CLARK [77], GOTTCHA [78], Kraken [79], and One Codex platform [59] have been developed to explore the taxonomic assignment and functional diversity of complicated microbial metagenomes.

Basing from the evaluation of the accuracy and speed of 14 metagenome bioinformatics tools by Lindgreen et al. [60], we also selected One Codex platform to analyze our metagenomic data and found that One Codex is much more effective and speedy than SOAPaligner (Table S9). Furthermore, taxonomic compositions at the genus level based on One Codex DB by One Codex were consistent with those results based on RefSeq DB by One Codex (Table S17).

Besides deep sequencing full-length 16S rDNA [76], PacBio SMRT has significantly improved the metagenome assembly when it is combined with HiSeq 2000 data [80]. A recent study has noted that MinION, a nanopore-based long-read sequencing platform, followed by Kraken or One Codex bioinformatics analysis, displays the potential to provide accurate and rapid metagenomic analysis [81]. This technology may be the most important advancement in environmental metagenome sequencing and functional discovery, because accurate, long (multi-kb) synthetic reads have detected rare microorganisms and resolved complex populations [82].

### 4.4. Potential Reason and Consequences of Depletion of Rahnella Genus

The previous studies have reported that several ways by which transgenic plants affected the structure and function of soil microbes, including horizontal gene transfer from transgenic plant to microbes, [83,84,85,86] transgene expression products released from roots or the residues of transgenic plants [6,87,88], and unintentional shifts of root exudate composition in rhizosphere soil of transgenic plants [89,90,91]. However, we feel it is very hard to analyze the main reason of depletion of *Rahnella*, because the male parent of the GT soybean line NZL02-92, which was the male parent of N698, was the derivative strain of the GT soybean line AG4501 that was a patented product bred by Asgrow Company, and to date, only a related reference is available from all the databases at Web of Science as follows: “Buettner MJ 1998. New soybean cultivar (9312069421822B) is useful in plant breeding programs to produce superior hybrids. Patent No. US5998704-A.9312069421822B has a higher yield, 73.06 bushels/acre compared to 68.53 bushels/acre for Asgrow AG4501.”

In turn, these affected soil microbes, especially rhizosphere microbes, could eventually have impacts on the growth and nutritional status of the transgenic plants. In this study, we found some dominant genera including *Rahnella* shown to be affected in the rhizosphere soil of the transgenic soybean plants. The potential consequences of depletion of *Rahnella* could affect the growth, pathogen suppression, and health of the transgenic soybean plant. Since some species or strains of the genus *Rahnella* have already identified as plant growth-promoting rhizobacteria, for example, some isolates of *R. aquatilis* that reportedly act as a nitrogen fixer [92,93]. Some strains or species of *Rahnella* genus have the phosphate-solubilizing traits, including a *R. aquatilis* strain ISL19 [94], and a *Rahnella* sp. strain isolated from *Hippophae rhamnoides* rhizosphere [95]. Furthermore, one strain of *R. aquatilis* was identified as an efficient phytate-degrading rhizobacteria [96]. Moreover, some strain or species of *R. aquatilis* can inhibit plant pathogen, including a *R. aquatilis* strain HX2 [97] and a *R. aquatilis* isolate 36 [98]. Additionally, the endophytic bacterium *Rahnella* sp. JN6 showed very high Cd, Pb and Zn tolerance and plant growth-promoting traits [99]. Thus, these microbial activities just mentioned above like nitrogen-fixing, phosphate-solubilizing, phytate-degrading, inhibition of plant pathogen, and bioremediation will undoubtedly be beneficial for the plant growth.

## 5. Conclusions

Consistent with our previous findings via 16S rDNA V4 region amplicon sequencing, this study showed that the GT soybean line N698 influences the rhizosphere bacterial communities at the flowering stage compared with the cultivar MD12 as control under field conditions via 16S rDNA V5–V7 region amplicon sequencing and shotgun metagenomic approaches. In specific, the GT soybean line N698 negatively affects *Rahnella*, *Janthinobacterium*, *Stenotrophomonas*, *Sphingomonas* and *Luteibacter* while positively affects *Arthrobacter*, *Bradyrhizobium*, *Ramlibacter* and *Nitrospira*.

## Figures and Tables

**Table 1:** Identification and comparison of special genera in the rhizospheric soil of the glyphosate-tolerant (GT) soy bean line N698 and its control MD12 by shotgun metagenomic approaches.

Bioinformatics Tool	SOAPaligner		One Codex Data Platform			
Database	NCBI nucleotide database		One Codex DB		RefSeq DB	
Sample name	MGMRh	MGNRh	MGMRh	MGNRh	MGMRh	MGNRh
Total mapped reads	790,310	318,187	7,413,129	6,118,123	4,878,689	4,058,863
**Genus name**	Mapped reads (proportions of total mapped reads)					
*Rahnella* ^1^	316,533 (40.052%)	385 (0.1210%)	363,110 (4.898%)	510 (0.00834%)	216,768 (4.443%)	364 (0.00897%)
*Serratia*	28,900 (3.657%)	38 (0.0120%)	36,565 (0.4932%)	1416 (0.02314%)	20,940 (0.4293%)	1959 (0.04826%)
*Yersinia* ^2^	381 (0.0482%)	6 (0.0019%)	337 (0.0045%)	37 (0.00060%)	1144 (0.0234%)	111 (0.00273%)
*Variovorax* ^1^	27,824 (3.521%)	21,698 (6.819%)	158,661 (2.1403%)	150,119 (2.4537%)	123,709 (2.536%)	109,798 (2.7051%)
*Ewingella* ^1^	ND	ND	1077 (0.01453%)	67 (0.00110%)	ND	ND
*Ramlibacter* ^1^	1846 (02336%)	2518 (0.7913%)	12,642 (0.1705%)	17,414 (0.28463%)	17,680 (0.3624%)	23,159 (0.57058%)
*Luteibacter* ^2^	24 (0.0030%)	2 (0.0006%)	129,081 (1.7413%)	9029 (0.14758%)	ND	ND
*Pedobacter* ^2^	638 (0.0807%)	166 (0.0522%)	29,399 (0.3966%)	6812 (0.11134%)	5210 (0.1068%)	2026 (0.04991%)
*Flavisolibacter* ^2^	20 (0.0025%)	19 (0.0060%)	ND	ND	ND	ND

^1^ Genus detected by 16S rDNA V5–7 region amplicon deep sequencing only. ^2^ Genus detected by 16S rDNA V4 region amplicon deep sequencing only. ^3^ ND means “not detected”. MGMRh and MGNRh represent pooled rhizospheric DNA sample of MRh and NRh for shotgun metagenome sequencing, respectively.

**Table 2:** Identification and comparison of rhizobacterial species of *Rahnella* and *Serratia* in MGMRh and MGNRh by shotgun metagenomic approaches.

Bioinformatics Tool	SOAPaligner		One Codex Data Platform			
Database	NCBI nucleotide database		One Codex DB		RefSeq DB	
Sample name	MGMRh	MGNRh	MGMRh	MGNRh	MGMRh	MGNRh
Total mapped reads	790,310	318,187	7,413,129	6,118,123	4,878,689	4,058,863
**Species name** ^1,2^						
*Rahnella aquatilis*	8260	9	26,556	46	216,768	364
*Rahnella* sp. WP5	ND	ND	89,230	86	ND	ND
*Rahnella* sp. Y9602	37,445	35	12,208	25	ND	ND
SUM	**45,705**	**44**	**127,994**	**157**	**216,768**	**364**
*Serratia liquefaciens*	24,395	25	32,253	91	ND	ND
*Serratia proteamaculans*	267	0	ND	ND	7693	184
*Serratia plymuthica*	258	1	304	37	6042	289
*Serratia marcescens*	41	4	452	149	4184	1223
*Serratia rubidaea*	ND	ND	142	134	ND	ND
*Serratia* sp. S4	ND	ND	229	0	ND	ND
*Serratia quinivorans*	ND	ND	227	0	ND	ND
*Serratia fonticola*	8	0	114	33	1047	134
*Serratia entomophila*	88	0	ND	ND	ND	ND
*Serratia odorifera*	65	0	83	54	ND	ND
*Serratia* sp. 506_PEND	ND	ND	102	0	ND	ND
*Serratia* sp. SCBI	52	2	15	0	ND	ND
*Serratia grimesii*	1	0	72	0	ND	ND
SUM	**25,175**	**32**	**33,993**	**498**	**18,966**	**1830**

^1^ Those values in bold plus labeled with red color represent the top species in the rhizospheric soil of the control cultivar MD12 (MGMRh). ^2^ Those species not listed in this table if their mapped read counts in two samples were less than 10 reads. ^3^ ND means “not detected”.

## Supplementary Materials

The following are available online at http://www.mdpi.com/2073-4425/9/4/214/s1, Figure S1: Rarefaction curves of alpha diversity indices of MRh and NRh samples (16S rDNA V5–V7), File S1: OTU_final sequence in MRh and NRh samples (16S rDNA V5–V7), Table S1: Summary of reads, tags and OTUs of MRh and NRh samples (16S rDNA V5–V7), Table S2: Normalized OTU table for biom format of MRh and NRh samples at the flowering stage (16S rDNA V5–V7), Table S3: Comparison of alpha diversity between MRh and NRh based on normalized OTU table (16S rDNA V5–V7), Table S4: Differentially relative abundances of bacterial phyla between MRh and NRh (16S rDNA V5–V7), Table S5: Absolute abundances of bacterial genera in MRh and NRh samples (16S rDNA V5–V7), Table S6: Differentially relative abundances of characterized genera between MRh and NRh (16S rDNA V5–V7), Table S7: Absolute abundances of bacterial species in MRh and NRh samples (16S rDNA V5–V7), Table S8: Top 30 dominant genera in MRh and NRh samples revealed by 16S rDNA amplicon deep sequencing, Table S9: Quality control report and statistical summary of assembled metagenome sequencing data (IDBA + One codex), Table S10: Comparison of mapped reads between MGMRh and MGNRh at all taxonomic levels by SOAPaligner, Table S11: Computation and comparison of mapped reads between MGMRh and MGNRh at all taxonomic levels by One Codex based on One Codex DB, Table S12: Computation and comparison of mapped reads between MGMRh and MGNRh at the genus level by One Codex based on One Codex DB, Table S13: Computation and comparison of mapped reads between MGMRh and MGNRh at all taxonomic levels by One Codex based on RefSeq DB, Table S14: Computation and comparison of mapped reads between MGMRh and MGNRh at the genus level by One Codex based on RefSeq DB, Table S15: Computation and comparison of mapped reads between MGMRh and MGNRh at the species level by One Codex based on One Codex DB, Table S16: Computation and comparison of mapped reads between MGMRh and MGNRh at the species level by One Codex based on RefSeq DB, Table S17: Comparison of mapped read count (%) of top 30 genera in the MGMRh and MGNRh samples by shotgun metagenomic approaches.
